# Price-Setting Power in Global Value Chains: The Cases of Price Stabilisation in the Cocoa Sectors in Côte d’Ivoire and Ghana

**DOI:** 10.1057/s41287-022-00543-z

**Published:** 2022-06-23

**Authors:** Cornelia Staritz, Bernhard Tröster, Jan Grumiller, Felix Maile

**Affiliations:** 1 Department of Development Studies, University of Vienna, Sensengasse 3/2/2, 1090 Vienna, Austria; 2 Austrian Foundation for Development Research (ÖFSE), Sensengasse 3, 1090 Vienna, Austria

**Keywords:** Global value chains, Price-setting power, Price stabilisation, Financialisation, Cocoa, Grinder-traders, Côte d’Ivoire, Ghana

## Abstract

This paper calls for integrating price-setting power and related uneven exposure to price risks into the analysis of governance in global value chains (GVCs) as it adds to other power dimensions in producing unequal distributional outcomes. This is shown for the cocoa GVC, in which—unlike in today’s mostly liberalised market structures—the world’s top cocoa-producing countries, Côte d’Ivoire and Ghana, pursue price stabilisation measures. These measures address intra-seasonal producer price volatility, and recent collaboration has achieved a living-income differential on top of export prices, but such measures do not shield export and producer prices from inter-seasonal variations in world prices determined on commodity derivatives markets. Based on interviews with actors along the cocoa GVC, we argue that this is related to the price-setting power of ‘grinder-traders’ and the key role of financial hedging and trading on commodity derivatives markets in their business strategies. Financialisation processes have increased derivatives trading’s complexity and short-termism, accelerating consolidation among grinder-traders and making price stabilisation more challenging. Through their price stabilisation systems, Côte d’Ivoire and Ghana have maintained some price-setting power in the cocoa GVC, but largely remain ‘global price-takers’, with prices determined on derivatives markets and transmitted along the cocoa GVC through grinder-traders.

**Supplementary Information:**

The online version contains supplementary material available at 10.1057/s41287-022-00543-z.

## Resumen

Cet étude lance un appel à l’intégration des pouvoirs en matière de fixation des prix, et les inégalités y associés dans l’exposition aux risque des prix, dans l’analyses de la gouvernance des chaines de valeur mondiales (en anglais: « global value chains», GVC), puisque cela se somme à d’autres dimensions du pouvoir, produisant des répartitions de résultats inégales. Ici on étude la GVC du cacao, où – a différence des structures des marchés pour la plupart libéralisées – les plus gros producteurs de cacao, le Cote d’Ivoire et le Ghana, poursuivent des mesures de stabilisation des prix. Ces mesures visent la stabilisation de la volatilité intra-saisonnières des prix aux producteurs (les collaborations récentes ont achevé un différentiel sur les revenus minimum et sur les prix d’exportation), mais ces mesures ne protègent pas les prix d’exportation et aux producteurs des variations inter-saisonnières des prix mondiales, déterminés par les marchés des instruments dérivés sur les produits de base. Nous basant sur des entretiens avec des acteurs dans la GVC du cacao, nous argumentons que cela est dû au pouvoir de fixation des prix des « broyeurs-négociants», et au rôle clé dans leurs stratégies d’entreprise et commerciales des couvertures financières sur les instruments dérivés liés à des produits de base. Les processus de financialisation ont augmenté la complexité et le court-termisme des transactions sur les produits dérivés, ce qui a accéléré la consolidation parmi les broyeurs-négociants, rendant la stabilisation des prix encore plus difficile. Grâce à leurs systèmes de stabilisation des prix, le Cote d’Ivoire et le Ghana ont retenu quelque pouvoir de fixation des prix dans la GVC du cacao; cependant ils demeurent des « preneurs de prix», les prix étant déterminés par les marchés des produits dérivés et transmis au long de la GVC du cacao par les broyeurs-négociants.

## Introduction

Commodity dependence remains a crucial development challenge for many lower-income countries, and commodity prices’ volatility and associated macroeconomic and structural vulnerabilities are at the centre of this challenge (UNCTAD and FAO 2017; Nissanke 2019). After renewed reform discussions in the context of the commodity price boom and bust in the late 2000s, the political will to address commodity price volatility in the form of price stabilisation mechanisms at the international level is again limited. In this context, it is important to understand the possibilities of producer countries to deal with commodity price volatility through national policies, as well as such interventions’ limitations, in light of how actual prices are set and transmitted in global value chains (GVCs). This paper aims to do this by calling for integrating price-setting power and related uneven exposure to price risks into the analysis of governance in GVCs based on an assessment of the cocoa GVC and price stabilisation measures in the top cocoa-producing countries Côte d’Ivoire and Ghana.

Public regulation has remained rather strong in tropical agriculture export sectors, particularly in Sub-Saharan Africa (SSA), given their economic importance and the high share of smallholder producers, but price stabilisation measures have played a minor role since the 1980s and’90s, when such instruments were largely dismantled in the context of structural adjustment programmes (SAPs). However, the world’s top two cocoa-producing countries—Côte d’Ivoire and Ghana, which accounted for 45 and 18% of global production in 2020 (ICCO 2021), respectively—are important exceptions (Gilbert 2009). Côte d’Ivoire re-regulated its system after a more liberalised period from the late 1990s to 2011–2012, whereas Ghana largely retained its regulatory system. In 2019, the two countries also started to collaborate with regard to price regulation by introducing a living-income differential (LID). This paper uses the cases of Côte d’Ivoire and Ghana to analyse price stabilisation measures’ possibilities and limitations, as well as related outcomes on intra- and inter-seasonal export and producer price volatility and the distribution of price risks in the context of inter-firm relations and price-setting institutions in the cocoa GVC. These issues are relevant for commodity export sectors beyond cocoa in Côte d’Ivoire and Ghana, particularly given the discussions in commodity producing countries on re-regulating liberalised market structures since the mid-2000s.^1^ Because of the concentration of global cocoa production, the challenges that Côte d’Ivoire and Ghana face in price stabilisation likely will be heightened in producer countries with comparatively less-powerful supplier positions.

This paper builds on the GVC approach and its foci on inter-firm governance in global production arrangements (e.g. Gereffi et al. 2001) by integrating price-setting power, i.e. the power to determine types of contracts and contractual terms around prices—including reference prices, discounts or premiums, time of price fixing and other price-related stipulations—into bilateral transactions. Price-setting power is crucial because these terms determine how price risks are distributed among actors in GVCs, adding to other power dimensions in GVCs. Thus, our conceptual approach emphasises firms’ price-setting strategies and price-setting institutions at different levels in commodity sectors. Commodity trading houses (CTHs) generally have important price-setting power given their intermediary position between exporters and downstream processors or manufacturers, as well as their concentration, particularly compared with exporters. CTHs use commodity derivatives markets for price risk management through financial hedging and for price benchmarks, transmitting these futures prices along GVCs. However, sector institutions that publish standardised contracts also may have price-setting power, as well as producer country institutions that impact how price risks are distributed among domestic actors (Bargawi and Newman 2017; Staritz et al. 2018).

Methodologically, this paper is based on an analysis of trade, sector and financial data and policy documents on price stabilisation regulations in Côte d’Ivoire and Ghana, as well as 47 semi-structured interviews with various actors along the cocoa GVC between 2017 and 2022. The interviews were conducted with representatives from five multinational chocolate manufacturers and ten CTHs—which we term *grinder-traders* in the cocoa sector, given their role in processing (grinding) and trading cocoa beans and intermediary products—in European headquarters and in subsidiaries in Côte d’Ivoire and Ghana. Among them are the top four international grinder-traders. We further interviewed representatives from six financial investors and two international cocoa sector associations: the International Cocoa Organization (ICCO) and the Federation of Cocoa Commerce (FCC). In Côte d’Ivoire and Ghana, we interviewed representatives from the two sector parastatals, two cocoa sector associations, four local traders, seven farmer associations or cooperatives and eight sector experts from donor, international and local civil society organisations and research institutes. Most interviews were conducted face-to-face, but some were conducted through phone or online video calls (see appendix for more details on the interviews conducted).

Our analysis shows that Côte d’Ivoire and Ghana have maintained some price-setting power through the use of forward contracts as the basis for fixed intra-seasonal producer prices. This shields producers from short-term volatility in world prices determined on commodity derivatives markets. However, inter-seasonal price variations are not reduced, with price risks between seasons largely borne by producers. Recent collaboration on price regulation between the two countries seems to have been successful in setting a higher export premium in the form of a LID, but it has not delinked export prices from futures prices. We argue that this is related to grinder-traders’ price-setting power in the cocoa GVC and their strong interest in futures-based prices, given their business strategies around commodity derivatives markets. While interrelated physical and financial strategies long have been at the centre of CTHs’ activities, financialisation processes have increased derivatives trading’s complexity and short-term nature, accelerating consolidation among multi-commodity CTHs and making price stabilisation more challenging. Reinforced by their dependence on foreign exchange linked to cocoa exports, Côte d’Ivoire and Ghana remain largely ‘global price-takers’, with world prices determined on increasingly financialised commodity derivatives markets and transmitted along GVCs through grinder-traders.

Section “Commodity Prices, Price-Setting and Global Value Chains” conceptualises price-setting in GVCs as a process determined by inter-firm relations and institutions at the global and producer country levels. Section “Price-Setting in Cocoa Global Value Chains” discusses key developments in the global cocoa sector related to price-setting institutions, grinder-traders’ strategies and financialisation processes. The next three sections analyse price stabilisation measures in Côte d’Ivoire and Ghana’s cocoa sectors and recent collaboration in the context of the LID, as well as outcomes from both in terms of price risk distribution, also in comparison with the other major West African cocoa-producing countries Cameroon and Nigeria. The last section concludes.

## Commodity Prices, Price-Setting and Global Value Chains

The relation between commodity dependence and economic development has been the subject of heated debate. For structural development economists, commodity price dynamics function as one key mechanism—and even arguably the key mechanism—that explains the negative impacts from commodity dependence on economic development. The Prebisch–Singer thesis (Prebisch 1950; Singer 1950) can be viewed as a starting point for this perspective, focusing on the secular decline of real terms of trade in commodities *vis à vis* manufactured goods.^2^ Furthermore, commodity price instability per se has been identified as a major concern. One literature strand has focussed on pro-cyclical consumption, investment and fiscal spending patterns linked to commodity price fluctuations, as well as related variations in terms of trade as the main causes of macroeconomic instability. Another strand has focussed on ‘Dutch disease’ effects and more generally on the negative effects of commodity dependence on manufacturing sectors. Initially introduced as a static concept focussing on exchange rate appreciation and ‘pull effects’ on labour and material inputs linked to commodity discoveries that increase production costs in non-commodity sectors, volatility in terms of trade and real exchange rates due to commodity price swings has been identified as impeding manufacturing sector development (Ocampo 2017; UNCTAD and FAO 2017).^3^

The focus on these macroeconomic and structural implications from commodity price dynamics generally comes at the expense of assessing specific commodity sector dynamics in terms of actual price-setting processes and institutions, and related outcomes at the commodity sector level. Older literature has analysed price stabilisation measures in specific sectors, particularly in the context of national marketing boards and international commodity agreements (ICAs) (for cocoa, see, e.g. Kofi 1976; Hecht 1983; Alence 2001; Losch 2002). These contributions demonstrate that commodity prices are not determined on abstract markets, but rather through different actors’ interactions and power relations in specific sectors—e.g. producers, traders, exporters, international buyers, governments and colonial administrations—embedded in political economic contexts and institutions at the global and producer country level. Such an approach corresponds with network and institutional approaches that view price formation as a political and contested process in which prices are the outcome of struggles between actors (Beckert 2011). Bargawi and Newman (2017) stated that ‘more powerful actors are able to impose their method of valuation on others and, thus, affect the distribution of value’ (178). The formation of a so-called ‘world price’ and how it appears in contracts on physical commodity transactions and subsequently affects prices and price risks for different actors are essential in these struggles (see also C̦alıșkan 2010).

A sector perspective that focusses on firm strategies and inter-firm power relations, as well as on institutions in which such relations are embedded, is provided through the GVC approach, which analyses the organisation and governance of global production and trade in specific sectors, particularly through lead firms’ role and how it affects producers’ development prospects (e.g. Gereffi et al. 2001). The GVC approach also has been applied to tropical agriculture sectors, including cocoa (Gibbon 2001; for cocoa, see, e.g. Fold 2002; Talbot 2002; Amanor 2012; Fold and Neilson 2016). These GVC studies are largely concerned with productivity and quality issues, particularly the deterioration in quality that many producer countries faced after liberalisation in the 1980s and’90s, along with producer price shares, as well as smallholder producers’ related upgrading and livelihood challenges. On the lead firm side, key research topics are concentration processes in GVCs and the resulting ‘buyer drivenness’, with lead firms such as coffee roasters and chocolate manufacturers increasingly focussing on branding, marketing and related quality conventions to shape the division of labour and entry barriers along GVCs.

However, GVC studies on tropical agriculture sectors generally have not focused on price-setting. The key characteristic of these products’ price volatility is acknowledged, but it is often not the analytical focus. Studies that have assessed recent price stabilisation measures in SSA agricultural export sectors have tended to focus on the producer country level, analysing national market structures, but not how they play out in GVC governance structures (for cocoa in Ghana, see, e.g. Kolavalli and Vigneri 2017; Quarmine et al. 2014). This is problematic because these studies at least implicitly tend to perceive producer country institutions as ‘producer price setters’, masking the transnational dimension of how prices are set. We argue that a GVC perspective on commodity price-setting is useful, requiring the integration of ‘price-setting’ power in the sense of being able to determine types of contracts and contractual terms around prices, including reference prices, i.e. what is the ‘world price’; discounts or premiums; time of price fixing; and other price-related stipulations in bilateral transactions as a crucial dimension of governance and power relations in GVCs. Our definition of *price-setting power* differs from the ability to determine actual prices, which refers to benchmark prices largely determined on commodity derivatives markets. The power to decide on types of contracts and contractual terms around prices is also crucial, as these terms determine how price risks are distributed among actors in GVCs (see also Bargawi and Newman 2017; Staritz et al. 2018; Purcell 2018).

Even though other actors generally are identified as lead firms in tropical agriculture sectors, e.g. coffee roasters or chocolate manufacturers (Gibbon and Ponte 2005), exports from producer countries largely are handled through an increasingly concentrated number of CTHs, which hold contracts with exporters, in which prices and related stipulations are determined. Thus, CTHs have important price-setting power that stems from their relations with exporters, in which CTHs often have oligopsonistic positions on the one side. On the other side, they deliver commodities and commodity-based products at the volumes, characteristics and times that (other) lead firms demand. This intermediary role and CTHs’ ‘low-margin, high-volume and high-velocity business’ (Gibbon 2014) lead to large price risks, making price risk management a core part of their business.

Since the establishment of commodity derivatives markets in the 1860s for grains and cotton, and later in the 1920s for other tropical agriculture commodities, commodity price-setting has been linked to these markets, allowing for financial hedging for price risk management (ICT 2001). The financialisation of commodity derivatives markets in the form of financial actors increasingly investing in derivative-based, and also physical, agriculture assets and (lead) commodity firms conducting more complex financial activities, in addition to financial hedging (Newman 2009; Burch and Lawrence 2009; Clapp 2014), has manifested the relationship between futures and physical commodity prices further. Most strongly, this blurring between physical and financial activities is evident with CTHs, which have had a long tradition in hedging and speculative trading (van Huellen and Abubakar 2021).

Sector institutions or associations that publish draft contracts or contract rules and guidelines – such as the FCC and the Cocoa Merchants’ Association of America (CMMA) for cocoa, the European Coffee Federation and Green Coffee Association for coffee and the International Cotton Association for cotton—can play a role in price-setting. Historically, producer countries have wielded collective institutionalised price-setting power through ICAs and national regulations. After the abolition of these institutions, producer countries still regulated national market structures, but only a few have pursued price stabilisation measures, which determine domestic prices and how price risks are distributed between domestic actors (for coffee, see Newman 2009 and Bargawi and Newman 2017; for cotton, see Staritz et al. 2018; for cocoa, see van Huellen 2015). However, these national institutions must be assessed in the context of price-setting power along GVCs.

## Price-Setting in Cocoa Global Value Chains

West Africa’s important role in cocoa production emerged during the first chocolate boom in the early twentieth century, when chocolate consumption in Europe and North America grew by a factor of 10 between 1900 and 1940. West African countries obtained a production share of almost 70% by 1930 (Poelmans and Swinnen 2016), with growth particularly strong in the Gold Coast (now Ghana). While British and, to a lesser extent, French colonial authorities initially tried to enforce the implementation of large estates, indigenous farmers’ successful resistance in favour of small-scale farming was viewed as a main driver of the expansion, in addition to the use of the more robust and productive Forastero cocoa bean type (Ross 2014; Clarence-Smith 2000; Dand 2011). Cocoa production also benefited from this smallholder production system when declining prices during the interwar period made cocoa production unattractive in Latin America’s more plantation-based systems (Kofi 1976; Hecht 1983). Côte d’Ivoire’s cocoa sector experienced notable growth only after attaining independence in the 1960s when national policies incentivised expansion of the sector (Hecht 1983). West Africa remains the dominant global region for cocoa production, with a 76% share of global production in 2020, followed by Latin America, with 18%, and Asia, with 6% (ICCO 2020).

### National and International Institutional Changes

At the producer country level, different regulatory frameworks played a role in internal and external cocoa marketing to stabilise prices of outputs and inputs for smallholder producers after World War II (WWII). In West Africa, price stabilisation was introduced under colonial rule during WWII in the context of high price volatility during the largely unregulated interwar period. In Ghana, this also was related to resistance to the oligopsony of British traders and related local struggles (known as ‘cocoa holdups’). However, European traders’ interests were still entrenched in the new institutions (Alence 2001). While Ghana and Nigeria established marketing boards with direct interventions in physical trade, Côte d’Ivoire and Cameroon operated price stabilisation funds based on the French *caisse* systems, leaving physical handling to private actors (Kofi 1976; Gilbert 2009). The stabilisation mechanisms largely remained after these countries attained independence. In Ghana, the price stabilisation mechanism secured public revenues that were used for general public expenses (as was also the case during the colonial period; Bauer 1954) and for industrialisation efforts, with party politics playing an important role (Whitfield 2018). In Côte d’Ivoire, revenue generation was also an important motivation, along with expansion of cocoa production (Hecht 1983).

**Fig. 1:**
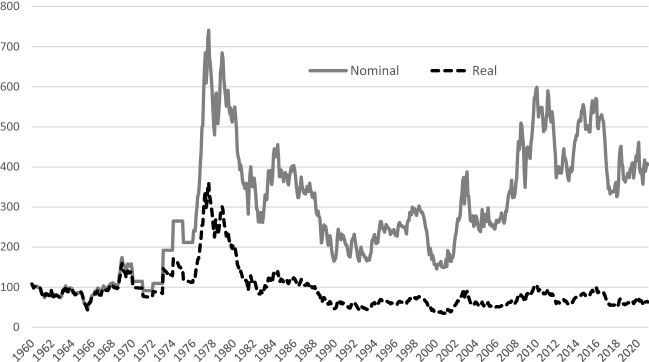
Real and nominal cocoa prices (index). ICCO prices deflated with US Producer Prices (All Commodities); Monthly average 1960 = 100. Source: World Bank, Federal Reserve Economic Data

National price stabilisation measures in the cocoa sector were accompanied by international regulations after WWII. This included the creation of the Alliance of Cocoa Producing Countries (COPAL) in 1962, comprising West African producers and Brazil, which aimed to coordinate national regulations and unsuccessfully attempted to increase cocoa prices in 1964–1965 by limiting supply (Hütz-Adams et al. 2016). In 1972, the International Cocoa Commodity Agreement (ICCA) was initiated to keep cocoa prices within defined price ranges through buffer stock interventions. However, the ICCA was not effective (unlike some other ICAs^4^) due to internal conflicts, lack of financial resources, initial non-participation by the US and Côte d’Ivoire’s temporary exit in the 1980s, as well as the rise of Indonesia as an important non-member producer country (Varangis and Schreiber 2001; Gilbert 1996). Throughout the 1970s, the world cocoa price remained above the price ceilings set, but this trend was reversed during the 1980s, when buffer stocks were unable to lift the world price above the set floor price (Gilbert 1996; see Fig. 1).

Also, an attempt by Côte d’Ivoire, which became the top producer country in the 1970s, to halt decreases in world prices through an export embargo from 1987 to 1989 (known as the ‘cocoa war’) was unsuccessful (Gombeaud et al. 1990; Losch 2002). The Ivorian *Caisse de stabilisation* (CAISTAB) engaged the French trader SUCDEN to store 200,000 tons of cocoa. However, most buyers anticipated this very costly stockpiling and waited until Côte d’Ivoire lifted the blockage in 1989, drawing on their own stocks or turning to other producer countries, such as Malaysia or Indonesia. Ivorian producers were the ‘forced funders’ of the embargo, given that they sold their beans at lower prices (Gombeaud et al. 1990). This demonstrated producer country governments’ limited power to control global exports, even in the case of the largest producer.

In the context of the non-functioning of the ICCA, price declines in the 1980s, financial constraints and rent extraction in national price stabilisation systems, and broader shifts towards market reform under the SAPs, state-controlled cocoa-marketing systems largely were abolished or reformed in the 1980s and’90s (Varangis and Schreiber 2001). Major donors considered liberalisation, i.e. changing the state’s role from a ‘marketer’ to a ‘regulator’ (Akiyama et al. 2001), as a way to increase transparency within sector institutions, lower marketing costs, raise farmers' incomes and increase competition through private operators (Varangis and Schreiber 2001). While Cameroon and Nigeria liberalised their marketing systems in the 1980s and’90s, Côte d’Ivoire and Ghana held on to their institutions, but reformed them, with Côte d’Ivoire abolishing price stabilisation from the late 1990s to the early 2010s. Through the lapse of the economic clauses, price regulation also was removed formally from the new ICCA in 1993 (Fold and Neilson 2016).

Continuing efforts to promote and (re-)regulate the national cocoa sector have been particularly pronounced in Côte d’Ivoire and Ghana, given their high dependence on cocoa. This dependence remains most significant in Côte d’Ivoire with 1.3 million smallholders in the cocoa sector (Hütz-Adams et al. 2016) and an export share of cocoa products, including beans and semi-processed products, in merchandise exports totalling 48% in 2020. In comparison, Ghana has around 800,000 cocoa smallholders, but the export dependence has decreased to 16% in 2020 because of gold and oil extraction since 2018 (UN Comtrade 2021). In other cocoa-producing countries in West Africa, Latin America and Asia, government intervention largely has been limited to productivity and quality improvements since the 1980s and’90s (Oomes et al. 2016).

### Grinder-Traders and Commodity Derivatives Markets

Through liberalisation in producer countries, transnational companies, i.e. chocolate manufacturers and grinder-traders, increasingly have dominated the cocoa GVC (Fold 2002). As chocolate manufacturers divested from producing semi-processed products (liquor, butter and powder) and related warehousing to focus on their higher-value core activities, the processing (grinding) and trading segments consolidated. Traders specialising in cocoa who traditionally acted as intermediaries between state-controlled exporters and chocolate manufacturers largely disappeared. Instead, large multi-commodity CTHs took over cocoa grinding and trading, using new processing technologies, their know-how in newly introduced bulk transportation of cocoa beans and their financial capacities to generate economies of scale and scope (Fold 2002). Considering that bulk cocoa can be transported in grain carriers, traditional grain traders, such as Cargill and ADM, entered the cocoa sector, allowing them to conduct business on both arms of the Europe-to-West Africa trip (Fold and Neilson 2016).^5^ Large cocoa grinders also merged with cocoa traders and integrated sourcing and trading activities (Fold and Neilson 2016). We term these actors *grinder-traders* because they fulfil a dual role in the cocoa GVC by buying cocoa beans from exporters, processing them and selling semi-processed products to chocolate manufacturers. Given their intermediary role and considering that both cocoa beans and semi-processed products are priced relative to futures prices (Araujo Bonjean and Brun 2016), price risk management is crucial for these actors, as with other CTHs.

Thus, the cocoa GVC today is characterised by two highly concentrated downstream segments, which has been described as ‘bipolar’ governance (Fold 2002); some scholars also refer to ‘tripolar’ governance given the increased role of retailers (Fold and Larsen, 2011). Since the 1980s, the *Big 6* chocolate manufacturers (Mars, Mondelez, Nestlé, Ferrero, Hershey and Lindt & Sprüngli) have dominated the production of chocolate products, accounting for 65% of cocoa consumption in 2016–2017 (Fold and Neilson 2016). After several large-scale mergers and acquisitions in the 2010s, the top four grinder-traders (Barry Callebaut, Cargill, Olam^6^ and Ecom^7^) accounted for 75% of global cocoa processing and trading in 2016–2017, with only two—Barry Callebaut and Cargill—being in charge of nearly half of the market (Fountain and Hütz-Adams 2018). The main grinder-trader factories are located in the Netherlands, Germany and France, and often are located near those of chocolate manufacturers, thereby generating economies of agglomeration in addition to economies of scale and scope (Araujo Beaujean and Brun 2016).

This concentrated structure on the buying side leaves cocoa exporter countries with few counterparts that have large-scale and often multi-commodity operations and have pursued increasingly similar price-setting and price risk management strategies that centre around derivatives markets. Commodity derivatives markets for cocoa were established in 1925 in New York and in 1928 in London during the first cocoa boom and have served as a reference point for world prices since then (ITC 2001).^8^ According to grinder-traders interviewed, prices of only a minimal share of cocoa—estimated at less than 5%—are delinked from futures prices, encompassing high-quality fine or flavoured cocoa that largely originates from Ecuador, the Dominican Republic and Peru^9^ and often is produced under traceable conditions (see also Fold and Neilson 2016). As a sector expert noted, *‘there is only marginal trade from origin to chocolate manufacturers, and the large entities buy and trade cocoa beans to feed their grinding facilities. They also have the financial power to serve the capital requirements to hedge with futures’.* Thus, large grinder-traders hedge all their physical transactions, whereas smaller traders have faced challenges to meet financial requirements for derivatives trading in addition to difficulties in pursuing high-volume bulk trade. These factors have contributed to consolidation and pushing remaining small actors towards niche markets (Gilbert 2009).

For hedging to be effective, prices in physical trade must reflect futures prices. Hedging requires taking a position in derivatives markets that opposes a physical position by holding the right (‘options’) or obligation (‘futures’) to buy or sell a physical commodity in the future at a given price. This allows for profits and losses from derivative and physical transactions to add up to zero and eliminate price risks. Price-setting practices that grinder-traders use in buying and selling transactions ensure these interrelations. After market liberalisation, transactions between grinder-traders and exporters moved generally away from ‘fixed-price-forward’ (or forward or outright) contracts to ‘spot price’ and ‘price-to-be-fixed’ (PTBF) contracts in producer countries without parastatals. While forward contracts can mitigate price risks for the duration of the forward contract by fixing prices when the contract is signed in advance of delivery, spot and PTBF contracts expose exporters to price variations because prices are fixed only at the time of delivery (which is the same as the time of signing the contract) for spot contracts or at the time of fixing (i.e. between the time of signing the contract and delivery and based on the counterpart’s decision) for PTBF contracts. Grinder-traders, like other CTHs, can integrate all three contract types into their price risk management as long as the reference price in physical contracts is based on futures prices. However, they generally prefer spot contracts on the buying side because they avoid counterparty risks.^10^ For exporters in producer countries, spot and PTBF contracts increase price risk exposure because prices are linked more closely to short-term price movements on commodity derivatives markets.

On the selling side, grinder-traders sell semi-processed products such as cocoa liquor, butter or powder. A leading grinder-trader interviewed summarises: ‘*On the buying side, we generally buy spot where there is no government involvement. And PTBF contracts are industry standard with chocolate manufacturers on our selling side*’. PTBF contracts ensure delivery of specified volumes of semi-processed products in advance, while prices are included as a ratio to futures prices but not determined yet. This enables chocolate manufacturers to lock in prices according to their own assessment of price developments and allows grinder-traders to hedge their price risks flexibly through futures contracts. As liquor is produced first, then either butter or powder, the price ratios for butter and powder are related and typically offset each other, i.e. combined butter/powder ratios remain relatively constant. Some grinder-traders also supply industrial chocolate, which is not priced relative to futures (Fold 2002; Araujo Bonjean and Brun 2016).

Since the 1990s, grinding increasingly has taken place in producer countries— particularly in Côte d’Ivoire, Ghana, Indonesia and Malaysia^11^—related to shifting GVC dynamics due to technological advances in transportation and producer country governments’ incentives (Grumiller 2018; Fold 2002; Gilbert 2009). However, this largely is done by grinder-traders that buy cocoa beans in producer countries, process them in their facilities in producer or consumer countries, and then trade processed products internally with their headquarters before they sell to chocolate manufacturers (van Huellen 2015). Thus, the key external transactions from a grinder-trader perspective, in which price-setting is relevant, entail buying cocoa beans from actors based in producer countries and selling semi-processed products to chocolate manufacturers.

Industry standards concerning contracts are important in terms of ‘working rules’, as van Huellen (2015) calls them, for actors engaging in physical trade. The two major trade associations in the cocoa market—the FCC for European markets and CMMA for North American markets—provide such contract rules, which generally are the basis for negotiations on actual contracts and for arbitration services (Dand 2011). The FCC currently has around 190 members, including cocoa grinder-traders and chocolate manufacturers, as well as financial actors, such as hedge funds and futures exchanges, along with producer country institutions from Ghana and Cote d’Ivoire. The FCC has drafted contracts and contractual rules for trade in cocoa beans, which are used in around 80% of the global cocoa trade, as they are the standard for West African exports. These rules are particularly important with regard to delivery/transport and arbitrage, as well as quality standards and payment terms, but they do not say much about price benchmarks and other price-related stipulations. The exception is PTBF contracts, in which the use of futures prices is recommended, given the higher chances for disagreements.

### Financialisation of Commodity Markets

Since their establishment in the 1920s, cocoa derivatives markets have served as a means for price discovery and hedging, and as a place for speculative activities (Close 1959; Hieronymus 1977). Derivatives markets for cocoa and other commodities experienced strong inflows of speculative funds, particularly in the 1970s (Labys and Thomas 1975; see Mallaby 2010 for an example of the Commodities Corporation Fund, arguably the first hedge fund) and 1980s, when tradeable commodity indices were launched (Berg 2011). Financial actors’ role grew in the 2000s as investment banks, hedge funds and institutional investors entered these markets on an even larger scale linked to deregulation in the EU and particularly the US, and the spread of electronic trading, which allowed for new, more complex and short-term trading strategies (Engel 2019). Between 1986 and 2005, open interest in New York cocoa derivatives, i.e. the number of futures and options contracts not yet settled, grew five-fold, but commercial traders, which use futures contracts for hedging, remained the dominant trader class, comprising 70% of open interest. However, since 2005, this share has decreased, and the further doubling in open interest has come primarily from financial actors. Disaggregated open interest data available since 2006 indicate that the share of the producer/merchant/processor/user (PMPU) category, comprising commercial traders, actually declined to 43% in total open interest and to 34% in long contracts’ open interest. Most open interest positions in 2020 were held by financial actors, including managed money (32%), swap dealers (also called index investors) (7%) and other reportables (14%) (CFTC 2021). Open interest data for London cocoa derivatives also indicate that financial actors hold a substantial share of open interest, but the average share of PMPUs with 67% remained higher compared with New York (ICE 2021).

Financial actors’ growing activities and dominance on commodity derivatives markets since the early 2000s, known as the ‘financialisation of commodities’, have triggered debate over their impact on commodity price dynamics (see, e.g. Ederer et al. 2016). Similar to other commodities, the empirical evidence for cocoa is mixed, as Haase et al. (2016) demonstrated in a meta-review. Cocoa often is included among other agricultural commodities in studies that look for commodity index investors’ impacts on commodity prices (Wimmer et al. 2021). Even though most of these studies find no general impact of index investor activities, several findings indicate a stronger effect on cocoa prices and other studies find impacts on cocoa futures returns (Sanders and Irwin 2017; Aulerich et al. 2013; Capelle-Blancard and Coulibaly 2011; Guilleminot et al. 2014), but Sanders and Irwin (2011) see a dampening effect from index investors on realised volatility. Van Huellen (2015) show that index investors impact the cocoa futures curve; thus, it can be a misleading indicator of fundamental demand and supply conditions. However, no extant studies exclusively have assessed the impact of money managers (particularly hedge funds) that generally pursue more short-term, long- and short-trend-following or algorithmic-based trading strategies on cocoa futures’ price dynamics.

In the context of increased volatility in cocoa futures prices since the mid-1990s, the interviewed sector experts and industry actors also view increased speed, complexity and short-termism of derivatives’ trading as a challenge for hedging, but also as an opportunity for actors with financial capacities. A top grinder-trader interviewed stated that *‘the role of other actors [on futures markets] using algorithms has grown (considerably), leading to much greater spikes, greater trading range (than) we have ever seen and greater dislocation from the fundamentals, which is challenging’.* Another grinder-trader interviewed added: *‘Things happen in cocoa prices that fundamentally make no sense—algorithms make that happen’.* However, he noted that this also ‘*provides liquid markets and presents opportunities for us’.* In particular, large multi-commodity grinder-traders, such as Cargill and Olam, have used these markets for financial business strategies, creating their own financial services units or hedge funds, investing on their own account, managing third-party money and selling investment products to physical and financial clients in the 2000s (Gibbon 2014; Murphy et al. 2012; Salerno 2016). However, most grinder-traders have closed or spun off their own funds in recent years and are focussing more on complex, structured and customised financial hedging services, as well as on over-the-counter markets that allow for more complex products and on market intelligence as part of their core business (Trafigura 2019; van Huellen and Abubakar 2021). A trader at a top grinder-trader explains it: ‘*We can offer price-insurance with profit opportunities to chocolate manufacturers on top of the physical sales. We build on PTBF contracts and integrate exotic derivatives that are increasingly traded over the counter in London and New York*’. These processes have contributed to further consolidation among grinder-traders, as well-developed financial units provide a competitive edge, compared with smaller traders with limited access to financial markets, information and resources (Newman 2009; van Huellen and Abubakar 2021).

## Price Stabilisation in Côte d’Ivoire and Ghana’s Cocoa Sectors

**Fig. 2:**
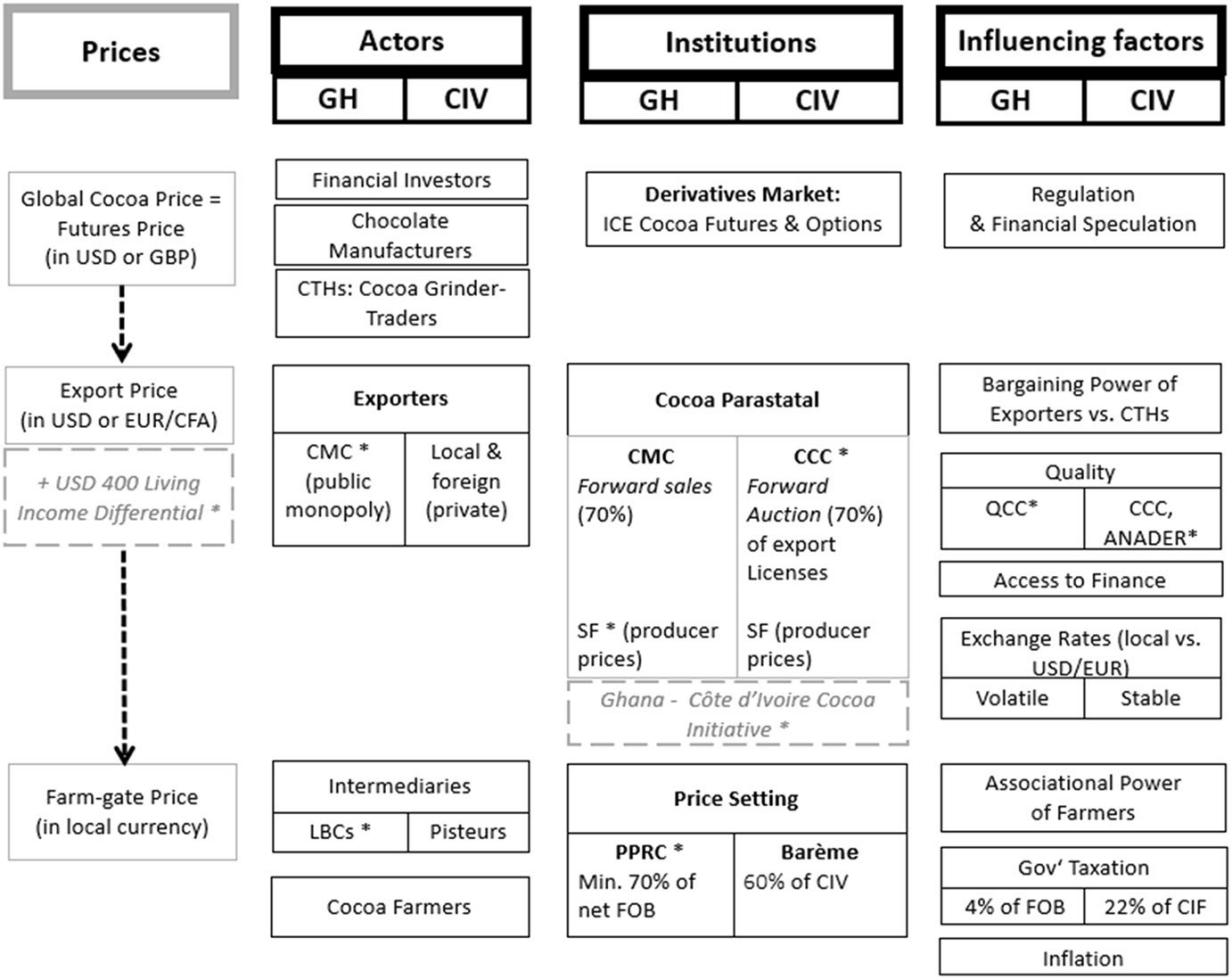
Cocoa price chain in Côte d'Ivoire and Ghana. * from season 2020/21 onwards (see Sect. 6); Anader: Agence Nationale d'Appui au Développement Rural, CCC: Conseil du Café-Cacao, CFA: West African CFA franc, CIV: Côte d’Ivoire, CMC: Cocoa Marketing Company, COCOBOD: Ghana Cocoa Board, CTHs: Commodity Trading Houses, EUR: Euro, GHA: Ghana, ICE: Intercontinental Exchange, LBC: Licensed Buying Company, PPRC: Producer Price Review Committee, QCC: Quality Control Company Limited, SF: Stabilisation Fund, USD United States Dollar. Source: Authors

Both major cocoa producer countries continue to regulate their cocoa sectors via parastatal institutions—the *Conseil du Café-Cacao* (CCC) in Côte d’Ivoire and the *Cocoa Marketing Board* (COCOBOD) in Ghana—and operate national producer price stabilisation mechanisms with the objective of protecting farmers from world price and exchange rate variations, as well as avoiding price differences between regions. Both countries can fix export prices for most of the cocoa bean harvest before the season via forward sales, providing the basis for a price schedule for each stage of internal marketing and exporting, as Fig. 2 shows. However, both countries historically have taken different approaches concerning the involvement of parastatals in the physical trade of cocoa beans, with COCOBOD being engaged in the physical handling and export of cocoa beans, unlike CCC. Concerning the constitution of cocoa exports, both countries have increased their share of origin grinding, which is related to industrial policies (Grumiller 2018). While in Côte d’Ivoire, the most important incentive is a reduction of the export tax (*droit unique de sorti*) for intermediate products, in Ghana, smaller (light) beans are sold at a discount to domestic grinders, effectively subsidising local grinding. In 2019, the share of cocoa beans in total cocoa-related exports accounted for 62% in Ghana and 72% in Côte d’Ivoire, with cocoa liquor accounting for 20% and 15%, butter for 13% and 9%, powder for 3% and 1%, and chocolate products for 1% and 3%, respectively (UN Comtrade 2021). Thus, relatively homogenous liquor and butter dominate processed exports, which also is related to the generally lower value of powder. For price-setting, this means that nearly all exports are priced relative to futures prices, as only chocolate products’ prices are delinked from futures prices.

### Ghana’s Price Regulation

The Cocoa Marketing Board, established in 1947, has regulated the Ghanaian cocoa sector by controlling internal and external marketing, setting producer prices and providing various services. The SAPs did not lead to the dismantling of the regulatory system, but with the reorganisation into COCOBOD, the objectives shifted from maximising tax revenues to linking producer to world prices and increasing farmers’ shares in export prices through lower marketing costs, which included substantial reductions in employees (Quarmine et al. 2014). The Producer Price Review Committee (PPRC) was established in 1983 and is responsible for negotiating producer prices among COCOBOD, farmers’ representatives and the Minister of Finance. Furthermore, the internal marketing system was deregulated partially in 1993 by introducing Licensed Buying Companies (LBCs) to procure cocoa beans from producers. This established an institutional mix: the public Produce Buying Company (PBC), which is the leading buyer, with a share of 30%, and around 30 private LBCs, including grinder-trader-owned LBCs, including the top four (Olam, ECOM, Cargill and Barry Callebaut), as well as Touton (Kolavalli and Vigneri 2017). However, the Cocoa Marketing Company (CMC), a subsidiary of COCOBOD, operates as a monopsony in the purchase of cocoa beans from LBCs and as a monopoly in the exportation of beans.

CMC sells around 70% of next season’s cocoa bean production through forward sales ahead of the harvest period. In the forward-selling process, CMC negotiates premiums with international buyers relative to underlying London cocoa futures prices. The forward sales largely determine the export price ahead of the harvest season and serve as collateral for an annual syndicated offshore loan, which provides working capital for LBCs to purchase cocoa beans and cheap access to foreign exchange for the government (Kolavalli and Vigneri 2017; van Huellen and Abubakar 2021). The remaining 30% is sold through spot contracts later in the season, exposing COCOBOD to the risk of price drops during the season.

The producer price for cocoa beans is fixed for one year at the beginning of the main crop season through the PPRC. Replacing a cost-based approach in 1998 that calculated producer prices based on expected costs (Quarmine et al. 2014), the ‘net FOB’ concept—i.e. the projected gross FOB export price (based on forward sales, estimates for the remaining spot sales during the season and projected average USD/GHC exchange rates), minus industry costs for services provided by COCOBOD—serves as the basis for negotiating income shares amongst farmers, LBCs, hauliers and COCOBOD subsidiaries. This pricing schedule links domestic prices to futures prices and sets minimum producer prices, maximum profit margins for LBCs and other actors in the domestic supply chain, and export duties. Today official policy consensus exists, in which farmers should receive at least 70% of the net FOB price. Ghana installed a stabilisation fund in 2004–2005 to support the intra-seasonal fixed producer price in case of an unexpected price drop in spot sales during the season. If realised export prices are higher than expected, COCOBOD also can pay a bonus to producers, which happened in 12 seasons between 2000–2001 and 2015–2016, but comprised only around 3% of producer prices (authors’ calculation based on Kolavalli and Vigneri 2017).^12^

### Côte d’Ivoire’s Price Regulation

CAISTAB regulated the Ivorian cocoa sector between 1955 and 1999, following the French model, in which private exporters played a more active role in internal and external marketing (Hecht 1983; Losch 2002). Producer prices were stabilised within and between seasons, but inter-seasonal price stabilisation was abandoned in 1990 after the failure of the ‘cocoa war’ (Benjamin and Deaton 1993). In the context of the SAPs, the system gradually was liberalised, culminating in the abolition of CAISTAB and intra-seasonal price stabilisation in 1999, though reform was very limited otherwise, and taxation remained at a high level. The sector’s disappointing overall performance led to calls for re-regulation in the 2000s, which was hampered by the civil war and political instability (Gilbert 2009). In 2011–2012, the Ivorian government finally established CCC as a regulatory body, which re-introduced a distribution system for export permits and a cost structure that fixes prices and margins for all domestic actors—called *barème*—in 2012–2013. CCC is not involved directly in the physical trade of cocoa beans, which is handled by around six dozen licensed exporters that source cocoa beans mostly through farm-gate (*pisteurs*) and wholesale (*traitants*) intermediaries. Grinder-traders are engaged in Côte d’Ivoire as exporters, as they took over many Ivorian firms during liberalisation in the 1990s (Losch 2002), increasingly sidelining locally owned exporters.

The *barème* is—similar to Ghana—based on fixed export earnings through forward sales. International buyers are required to buy 70–80% of the expected crop forward; the rest is sold through spot contracts during the season. Exporters acquire export permits (*déblocage*) from CCC in an auction that takes place twice a day, allowing them to export and source a specified quantity of beans at harvest time. CCC sets an auction reference price based on the London cocoa futures price adjusted to the ‘origin differential’ and the exchange rate conversion from GBP to CFA as the basis for the bidding process with exporters.^13^

The average price for export licenses realised in the auction (*prix de déblocage*) and projections on the remaining spot sales and exchange rates are the basis (*prix CAF de reference*) for calculating prices for different actors and taxes in the *barème*. This is set twice a year before the main and minor crop seasons in the context of stakeholder negotiations that CCC dominates. The agreed-upon policy goal is to fix the minimum producer price for farmers at 60% of the CIF reference price, but not below 50%. Private exporters’ involvement in the Ivorian system requires a compensation mechanism because they enter into export contracts with different prices, but source beans at a fixed price. With this compensation mechanism, exporters receive or need to pay compensation payments depending on whether they benefitted or lost due to the difference between the individual *prix de déblocage* and the general *prix CAF de reference.* The fixed producer price is stabilised further through a stabilisation fund in case spot sales during the season lead to lower-than-expected export earnings (KPMG 2018).

## Price Stabilisation’s Effects and Limitations in Côte d’Ivoire and Ghana

**Fig. 3:**
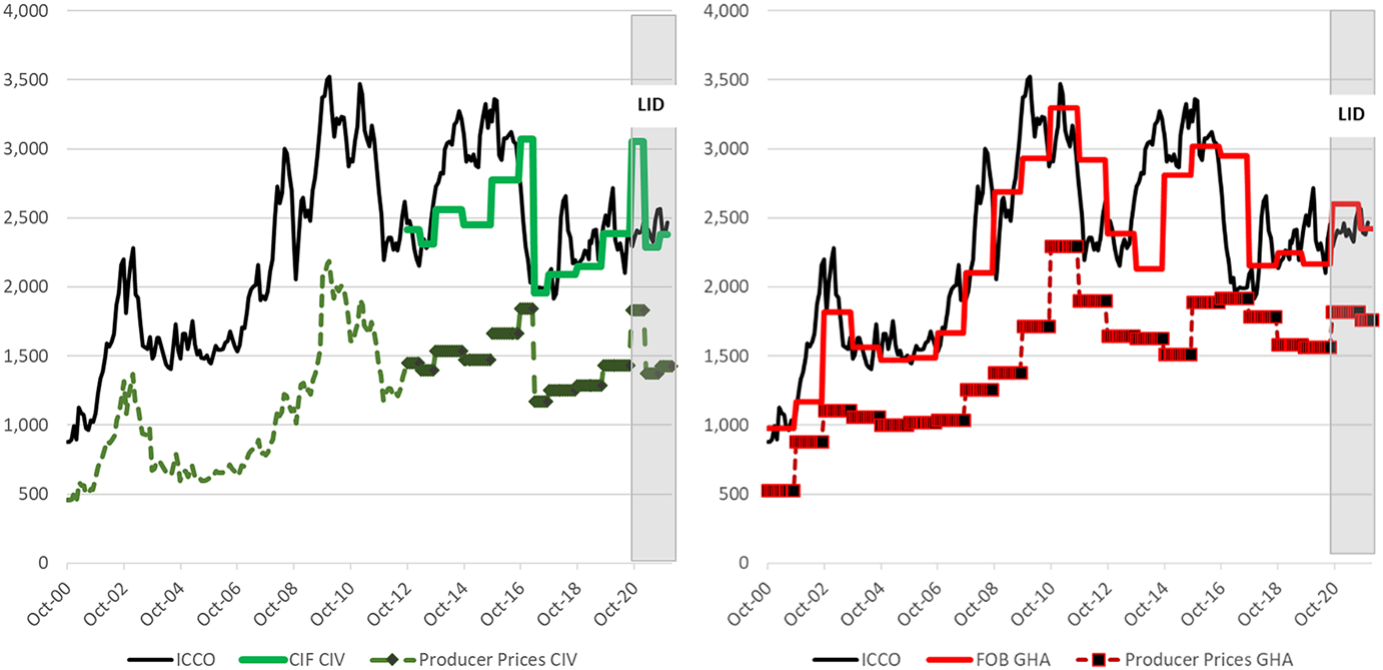
World cocoa, export and producer prices in Côte d’Ivoire and Ghana. Prices in USD. Basis for export prices are producer prices in local currency, converted to USD based on monthly USD/CFA average (Côte d’Ivoire) or GHS/USD in pricing formula (Ghana). Export prices in Côte d’Ivoire are estimated as a ratio of 1.67 to producer prices as producer prices are around 60% of CIF prices. In Ghana export prices are based on actual data on prices or shares of producer prices to FOB prices. Source: ICCO; World Bank; COCOBOD; CCC

Figure 3 shows the development of world cocoa prices (ICCO prices based on futures prices), as well as realised export and producer prices, in Côte d’Ivoire and Ghana between 2000–2001 and 2021–2022 in US dollars. Export prices in Ghana were stable within a season over the entire period. In Côte d’Ivoire, intra-seasonal price stabilisation started in 2012–2013, but export prices were adjusted downwards in the mid-seasons in 2012–2013 and 2016–2017. Export prices in both countries fluctuated between seasons, in which they followed the development of futures prices with a lag, as forward sales are based on futures prices before the season. Even though currency fluctuations also affect export earnings,^14^ futures prices are the major factor driving seasonal export prices. Correlation coefficients between year-on-year changes of average ICE London futures prices in the six months ahead of the marketing year and year-to-year changes in export prices accounted for 0.93 between 2000–2001 and 2018–2019 in Ghana and for 0.88 since the re-regulation in Côte d’Ivoire in 2012–2013. This is comparable to the liberalised cocoa market structures in Nigeria and Cameroon, where the correlation coefficients between futures prices and export prices during the same period (as these countries do not sell forward) accounted for 0.92 and 0.89, respectively, between 2000–2001 and 2018–2019 (ICE 2021; Eurostat 2021).

Thus, forward sales ensure stable intra-seasonal export prices to a large extent, but inter-seasonal export prices follow futures prices. Forward sales beyond one season could smooth inter-seasonal export prices, but they are hardly used. The Ivorian CCC stated that it aimed to sell forward up to 24 months, but grinder-traders preferred shorter forward contracts given that they typically hedge with futures contracts with delivery of up to six months. Furthermore, very little liquidity exists beyond near-term contracts (see also KPMG 2018; van Huellen 2015). Forward sales based on the near-season harvest also are required for access to foreign reserves, credit and trade finance, limiting the ability to adjust the time of sales, particularly regarding access to a syndicated loan in Ghana. Stabilisation funds in both countries theoretically could be used for inter-seasonal price smoothing, but their *raison d’etre* (and their size) are targeted towards intra-seasonal producer price stabilisation if export revenues (due to lower-than-expected prices for the remaining spot sales) fall short of expectations.

Neither parastatal generally uses financial hedging instruments to deal with price risks. They could do that in principle, even though it would be more complicated in the Ivorian system. However, beyond substantial knowledge, expertise and networks, which at least CMC traders have due to rigorous training in derivatives trading (van Huellen and Abubakar 2021), hedging requires access to large financial resources in US dollars and British pounds, given the large volumes of cocoa beans that the two countries export. This is related to security margins that must be deposited when buying or selling futures contracts, which are adjusted on a daily basis to futures price movements (so-called margin calls). Financialisation processes in derivatives markets, with their associated increase in short-term trading practices and price variations, have increased the financial costs of hedging, owing to more frequent and unpredictable margin calls. An alternative to futures would be the use of options, but those require a premium that can be more expensive than margins on futures (ITC 2001). COCOBOD representatives do not view hedging with financial derivatives as their core business and until now largely have refrained from accepting grinder-traders’ offers to create customised hedging products for them (see also van Huellen 2015). However, in recent years, COCOBOD experimented with options, but related losses reaffirmed their scepticism (COCOBOD 2017), particularly as emphasised by an interviewed CMC manager who noted that *‘these losses are hard to justify to the public’.*

Producer prices are linked to forward sales, as they are determined as a share of projected export earnings. Ghana aims for a 70% share of producer prices relative to the net FOB price, but in negotiations within PPRC, adjustments to this share have been used to address inter-seasonal price instability in producer prices (COCOBOD 2017). For instance, changes in producer prices have been less pronounced in years with lower export prices since 2015–2016, as producer price shares increased during these years. COCOBOD subsidised higher relative producer prices through so-called ‘cocoa bills’ (IMF 2019). COCOBOD could issue such bills given its direct access to cocoa supply and its financial autonomy, rendering it independent of the government budgeting process. In Côte d’Ivoire, the institutional framework—based on private actors physically exporting cocoa beans—is more rigid when it comes to producer price stabilisation. No possibilities exist to isolate producers from inter-seasonal world price fluctuations because export revenues are distributed as fixed margins to the private actors involved, with the only variable left to adjust being taxes However, the possibility of using taxes as a buffer for inter-seasonal producer price stabilisation is restricted because CCC has no autonomy to set these tax rates, and public revenues are heavily dependent on these taxes, i.e. governments have limited income precisely when prices are low. The only exception so far has been the reduction in export duties in mid-season 2016–2017 to keep the producer price share at 60% of the CIF export price (Aboa 2017).^15^

Thus, price stabilisation’s key impact in the two countries is intra-seasonal producer price stabilisation, and in Ghana, it also limits to a minimal extent inter-seasonal price volatility, based on the possibility of selling forward due to centralised marketing institutions. In this regard, Côte d’Ivoire and Ghana’s parastatals have maintained some price-setting power *vis a vis* grinder-traders. Grinder-traders interviewed stated that they accept forward sales because CCC and CMC can guarantee high volumes and quality *‘even though outright buying also means that you have to be prepared for high margin calls if prices change strongly, which can make it more costly for us*’ (top grinder-trader). This contrasts with liberalised systems, in which grinder-traders generally buy spot, as a grinder-trader noted: ‘*In other countries* [besides Côte d’Ivoire and Ghana], *we do not buy outright from an origin producer or trader who might have their own problems down the line … most is bought on spot basis’.* This is also the case in Cameroon and Nigeria, which have no centralised marketing institutions. Producers or producer organisations sell to local traders and licensed buyers that sell to exporters based on spot transactions, with exporters selling to or being owned by grinder-traders without any stabilisation of exports or producer prices (Oomes et al. 2016; Hütz-Adams et al. 2016). Thus, producer prices can vary substantially in the short-term and between farmers and regions, with farmers exposed to intra- and inter-seasonal price volatility (FAO and BASIC 2020; Hütz-Adams et al. 2016).

Aside from intra-seasonal price stabilisation, cocoa beans from Côte d’Ivoire and Ghana are sold with premiums (‘origin’ or ‘country differentials’) that are higher than in Nigeria and Cameroon, which also is related to some price-setting power. For example, export prices to the EU were 12% and 6% on average, respectively, higher for Ghana and Côte d’Ivoire compared to Nigeria and Cameroon between 2000 and 2018 (Eurostat 2021). This is related to quality differentials and the centralised quality control systems, which ensure that beans maintain a uniform, consistent and high quality, particularly in Ghana (Quarmine 2014), as well as to counterparty reliability. A key factor for the lower quality in the liberalised systems is the large share of informal marketing. For example, in Cameroon, 70% of exported cocoa is purchased from small traders (coaxers) at the farm-gate (Nkouedjo et al. 2020; Levai et al. 2015). However, higher premiums also are associated with consolidated sales channels that ensure beans are not sold below market prices. Thus, even though premiums depend on quality and reliability, exporters need to ensure that they get related premium, which is easier in centralised or regulated sales channels in which bargaining power is larger *vis a vis* international buyers.

Nevertheless, both countries are highly dependent on cocoa exports and related foreign exchange income, limiting their bargaining power *vis a vis* grinder-traders, which are increasingly concentrated. In Ghana, the number of CMC trading partners decreased from about 100 in 2000 to 11 in 2013 (van Huellen 2015). In Côte d’Ivoire, grinder-traders own the top five exporters—Cargill, SUCDEN, Touton, Olam and Barry Callebaut—and bought 80% of the export contracts during the 2018–2019 season (Aboa 2019), which is the same share that the top 10 bought in 2010–2011 (Araujo Bonjean and Brun 2016). Grinder-traders not only have dominant positions as buyers, but they also are involved in internal marketing, particularly in Côte d’Ivoire, where they also act as exporters in external marketing. Smaller independent exporters, particularly those based in producer countries, are at a disadvantage to large grinder-traders because they lack not only economies of scale and scope, but also have inferior access to low-cost financing, as well as financial hedging and trading on derivatives markets (see also Hütz-Adams et al. 2016).

**Fig. 4:**
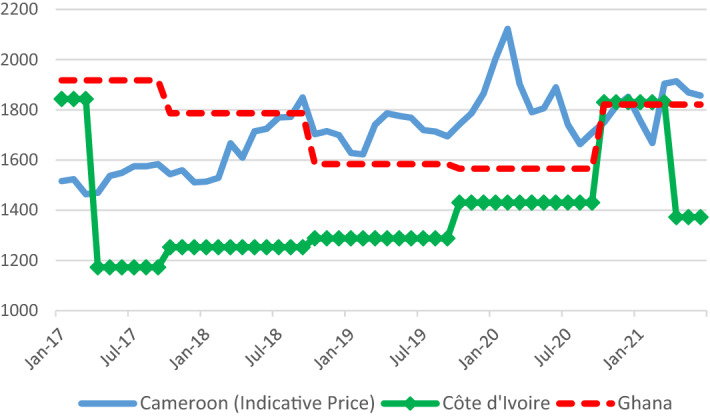
Cocoa producer and indicative minimum prices in Cameroon, Côte d’Ivoire and Ghana. Prices in USD. Producer prices in Côte d’Ivoire and Ghana in local currency, converted to USD based on monthly USD/CFA average (Côte d’Ivoire) or GHS/USD in pricing formula (Ghana). Indicative minimum prices in Cameroon are available from Jan-2017 to Jun-2021 (https://oncc.cm/cocoa-statistics) on a monthly basis and are converted to USD based on monthly USD/CFA averages. The actual producer prices in Cameroon differ and are generally lower. *Sources: ONCC; COCOBOD; CCC*

While intra-seasonal price stabilisation and higher country differentials on export prices are advantages of Côte d’Ivoire and Ghana’s systems, regulated systems typically are associated with lower shares of producer prices relative to world prices (see, e.g. Oomes et al. 2016). Compared with monthly ICCO prices, producer prices in Côte d’Ivoire reached a share of 57% of ICCO prices since price stabilisation in 2012–2013 and before the introduction of the LID in 2019–2020. In Ghana, producer prices totalled 68% of ICCO prices during this period. Although Cameroon’s Office National du Cacao et du Café (ONCC) publishes indicative minimum producer prices on a daily basis at around 75% of FOB prices (Oomes et.al. 2016; ONCC 2022), average producer prices have corresponded to only 66% of FOB export prices since 2010 and—given the discount in export prices *vis a vis* ICCO prices—to 61% of ICCO prices. This ratio appears to have deteriorated in recent years (FAO and BASIC 2020). Thus, producer price shares in Cameroon are actually lower than in Ghana—and not substantially higher than in Côte d’Ivoire—and more volatile, as shown in Fig. 4 for Cameroon’s indicative minimum prices. Comparable data unfortunately are not available for Nigeria.

Key factors that explain producer prices in regulated systems are taxes and other costs for parastatals. In Côte d’Ivoire, taxes accounted for 17% of export prices compared with only 4% in Ghana in 2017/2018 (FAO and BASIC 2020), but the net FOB calculation also includes costs for services that COCOBOD provides. Thus, the higher-quality premiums in Ghana are not translated fully to producers because costs for input provision and quality control are removed from export prices. However, the partial reinvestment of taxes and duties into the cocoa sector reduces production costs for farmers. It is estimated that the share of input costs on cocoa revenue is substantially lower for farmers in Ghana (15%) and Côte d’Ivoire (less than 30%) than those in Cameroon (35%), even though these government programmes do not benefit all farmers equally (Oomes et al. 2016; World Bank 2017).

Price stabilisation systems’ efficacy also depends on their resilience, which is limited, particularly in Côte d’Ivoire. The focal point in both price stabilisation systems is reliable estimations of export revenues, which serve as the basis for producer prices. Certain production-related risks can lead to over- or under-selling of forward contracts, as well as politically motivated risks, particularly in Ghana, where depreciation of the cedi and depleted foreign reserves led to overselling during the 2014–2015 season. However, the risks related to world price fluctuations for remaining spot sales during the season and forward sales performance are most important. Ghana’s system is more resilient in this respect because CMC has more control over cocoa supply and direct relationships with buyers, allowing for re-negotiation during difficult circumstances (see also Kolavalli and Vigneri 2017). In the Ivorian system, the stabilisation mechanism depends on private actors’ ability and willingness to comply with regulations. In particular, the likelihood of default on forward contracts is higher, which can be exemplified through the sharp world price drop of 35% from June 2016 to May 2017, which coincided with illicit speculative behaviour among local exporters in Côte d’Ivoire and consequent defaults on their forward contracts.^16^ CCC incurred a loss of CAF 199 billion (USD 360 million) (KPMG 2018) and lowered producer prices from CAF 1,100 to CFA 700 in mid-season. COCOBOD also faced challenges, and the stabilisation fund in both countries was not sufficient to cover the price drop. However, COCOBOD could still keep producer prices stable by issuing cocoa bonds, given its financial autonomy. However, the related debt burden’s sustainability is questionable, with COCOBOD’s outstanding, unsecured debt increasing to more than GHC 6 billion (IMF 2019). Thus, the world price drop in 2016–2017 demonstrates the limits of national price stabilisation systems, in which producer countries cannot influence the setting of world prices (i.e. futures prices) used as a reference for domestic prices.

## Abidjan Declaration

In the aftermath of the strong world price decline in 2016–2017, bilateral cooperation in the cocoa sector between Côte d’Ivoire and Ghana reached high-level political momentum, and both countries’ presidents signed the Abidjan Declaration in March 2018. In the centre of the declaration is a common strategy towards increased producer prices and related harmonisation of price regulation. Both governments aimed to set export prices directly by introducing a minimum export price. In June 2019, COCOBOD and CCC announced a common floor (FOB) export price of USD 2,600 per ton for the 2020–2021 crop season, in which farmers received 70%, or USD 1,820 per ton (COCOBOD 2019), raising producer prices significantly compared with recent years. Such a minimum export price, regardless of prevailing futures prices, would be a major deviation from previous price-setting practices.

Several sector experts interviewed indicated that international buyers resisted a minimum export price delinked from futures prices; thus, at technical meetings during summer 2019 between government officials and international buyers, including the top four grinder-traders—Barry Callebaut, Cargill, Olam and ECOM—and chocolate manufacturers such as Hershey and Mars, it was agreed that export prices would be determined as before—based on futures prices and ‘origin differentials’, but that buyers would pay an additional LID of USD 400 per ton, starting in the 2020–2021 season (FCC 2019). Nevertheless, COCOBOD and CCC held on to the target of paying 70% of the floor export price of USD 2,600, or USD 1,820 per ton, as a minimum producer price independent of the actual export price. Thus, if export prices, including LID and origin differentials, fall below USD 2,600 per ton, the parastatals or governments must pay the gap. A representative of a farmer-based organisation in Ghana noted: ‘*First it was said that buyers pay at least 2,600* [USD per ton]*. Now it is ‘business as usual’ plus 400, and COCOBOD (and) CCC pay the potential gap’.* This could exacerbate resilience problems in systems, even though both countries agreed to set up an additional stabilisation fund for this case that a common Ghana-Côte d’Ivoire Cocoa Initiative Secretariat would administer.^17^

Thus, the two countries were successful in setting a higher premium in terms of the LID, but futures prices remained the central reference in the price-setting mechanism. However, scepticism also exists regarding the premium. According to Aboa and Angel (2019), some international buyers reduced their forward purchases after the announcement of the LID and renegotiated the ‘origin differential’, arguing that it already is included in the LID.A Ghanaian researcher interviewed stated that grinder-traders ‘*mostly accept the LID, but the pressure on country differentials has increased in the ongoing forward sales*’. In 2021, COCOBOD and CCC officials claimed that international buyers were not paying country premiums, which almost offset the LID-generated premiums (Aboa 2021). Furthermore, an interviewed sector expert noted that ‘*there is (a) risk that the world price will adjust to this [LID] premium; the world price drops so far that ultimately, the world price plus the premium will make up roughly what would be paid without the premium anyway*’.

Regarding producer prices, both parastatals fixed producer prices at USD 1,820 per ton (1,000 CFA per kg in Côte d’Ivoire and 660 cedi per kg in Ghana) for the 2020–2021 season in October 2020, in line with their initial agreement. However, the COVID-19 pandemic magnified both systems’ vulnerability. With decreased cocoa demand and highly volatile futures prices, both countries faced challenges in securing forward contracts for the 2020–2021 season, and Côte d’Ivoire had to adjust the producer price down to USD 1,350 per ton for the mid-season starting in April 2021 (Reuters 2021). For the current 2021–2022 season, the Ivorian producer price was lifted slightly to USD 1,410 per ton, equivalent to producer prices before the LID (CCC 2021). Thus, the envisioned minimum farm-gate price of USD 1,820 per ton could not be maintained in Côte d’Ivoire. COCOBOD in Ghana announced that it would keep producer prices constant at 660 cedi per kg for the 2021–2022 season, thereby maintaining prices in the local currency. However, how long they can afford to subsidise this price is questionable (COCOBOD 2021).

## Conclusion

Market liberalisation in producer countries during the 1980s and ’90s has increased cocoa producers’ exposure to variations in world prices set on commodity derivatives markets. Price-setting power has shifted further towards grinder-traders, which intensified their business strategies around derivatives markets, binding futures prices more closely to physical contractual arrangements. Financialisation processes, leading to increased entry barriers around financial risk management, have contributed to the consolidation of grinder-traders, thereby limiting producer countries’ ability to pursue alternative selling arrangements and making price stabilisation more challenging. Unlike other cocoa-producing countries with liberalised market structures, the world’s largest cocoa-producing countries, Côte d’Ivoire and Ghana, have maintained some price-setting power through their state-controlled marketing systems in terms of price premiums and, most importantly, contractual arrangements (forward contracts), allowing them to stabilise intra-seasonal producer prices and shield producers from short-term price volatility. However, export and producer prices are stabilised for only one season, leaving producers affected by inter-seasonal price volatility. Recent collaboration has achieved setting a higher export premium in the form of a LID, but the distributional outcomes and the system’s resilience are questionable. Thus, despite powerful supplier positions, Côte d’Ivoire and Ghana remain largely ‘global price-takers’, with world prices determined on derivatives markets and transmitted along GVCs through grinder-traders. Price-setting power is likely even more limited for other producer countries with less-dominant positions and similar dependence on commodity export earnings.

Analysing the cocoa GVC and the two top producer countries’ price stabilisation measures, this paper argues for the importance of integrating price-setting into the analysis of governance in GVCs. Price-setting power and related uneven exposure to price risks and possibilities to deal with these risks add to other power dimensions in producing asymmetric relationships and unequal distributional outcomes in GVCs. It also demonstrates the importance of assessing price-setting along the entire GVC and how selling and buying prices and related price-setting strategies and institutions are interconnected, limiting the scope for price-setting in domestic commodity sectors. Price stabilisation measures in Côte d’Ivoire and Ghana have achieved intra-seasonal producer price stabilisation, which is an important outcome for producers compared with liberalised systems and one that has not necessarily come with lower producer price shares, but inter-seasonal price risks remain with producers, as well as the link between domestic prices and futures prices. Thus, national regulations can address distributional outcomes in terms of price risks and shares in producing countries to some degree. However, national structures alone cannot address price-setting power and related inequality in GVCs, driven importantly by CTHs and their links to commodity derivatives markets. Eventually, increasing producer countries’ price-setting power will require reducing their dependence on commodity export earnings, as well as on CTHs, which has become even more challenging in financialised commodity GVCs.

## Supplementary Information

Below is the link to the electronic supplementary material.

Supplementary file1 (DOCX 26 KB)

## Footnotes

1 For unsuccesful attempts to re-introduce price stabilisation in the cocoa sector in Cameroon and Nigeria, see Hütz-Adams et al. (2016); for continuing price stabilisation measures in Burkina Faso’s cotton sector and the reinstatement of such measures in Mozambique, see Staritz et al. (2018).

2 A large body of empirical literature is available on the Prebisch-Singer thesis, with some authors supporting and others questioning its validity (e.g., Hallam 2018; Baffes/Etienne 2016; Erten/Ocampo 2013).

3 Even though the adverse development effects of commodity dependence have been attributed in particular to ‘point resources’, such as oil and minerals, countries dependent on agricultural cash crops (‘diffuse resources’) also have faced these challenges, either because they are grown as plantation crops or because the state plays an important role in controlling and taxing the sector (Isham et al. 2005).

4 In the coffee sector, the related ICA was more effective with commodity-derivatives markets largely losing its role as a price benchmark in the 1960s (Talbot 2002).

5 Cargill integrated cocoa processing by taking over Gerkens in 1986 (https://www.cargill.com/food-beverage/na/gerkens-cocoa-powder) and ADM by taking over De Zaan in 1996 (https://fd.nl/frontpage/ondernemen/1085146/cacao-de-zaan-krijgt-moeder-uit-singapore).

6 Olam acquired ADM’s chocolate business in 2014–15 (McFarlne/Hunt 2015).

7 Ecom became a top grinder-trader when it took over Armajaro in 2013 (https://www.comunicaffe.com/global-ecom-agroindustrial-acquires-armajaro-trading-limited/).

8 The Intercontinental Exchange (ICE) operates both today. ICE London futures represent delivery in Northern Europe and serve as a reference price for cocoa from the SSA, while ICE New York futures represent delivery to the US East Coast, serving as a reference price for cocoa from Latin America and Asia.

9 https://www.icco.org/fine-or-flavor-cocoa/

10 Futures prices generally are adjusted through premiums or discounts related to quality or other commodity characteristics. However, with cocoa, they are relatively low, as bulk cocoa is a highly homogenous commodity, which reduces basis risk that cannot be hedged.

11 Today, Malaysia does not produce cocoa beans anymore and is technically not a producer country.

12 COCOBOD may adjust producer prices mid-season, but this is rarely the case due to the stabilisation mechanism and pressure from smallholders.

13 CCC does not share the detailed method of the auction reference price calculation.

14 ICE futures in London are quoted in British pounds, while Ghana sells in US dollars and Côte d’Ivoire in euros.

15 The CFA link to the euro eliminates exchange rate risks in Côte d’Ivoire. In Ghana, producer prices are kept stable in Ghanaian cedi throughout the season, as the projected gross FOB export price includes a conversion from US dollars to cedi. However, from season to season, producers also bear exchange rate risks.

16 These exporters acquired export licenses through the auction and presented counterfeit fixed-price contracts, but their contracts were PTBF with their buyers, resulting in lower selling than purchasing prices when prices fell. After this, exporters were required to validate that their export permits were linked to physical transactions by presenting counterparty forward contracts with buyers and by depositing 2.5% of the export value as collateral (KPMG 2018).

17 For periods when export prices are higher than USD 2,600 and up to USD 2,900 per ton, the additional revenue would be used for bonus payments to farmers or investments in the sector, while price increases beyond USD 2,900 per ton would flow exclusively into the stabilisation fund (FCC 2019).

## References

[CR1] Aboa, A. 2017. Ivory Coast to Lift Cocoa Farm Price, Hold Export Tax. https://www.reuters.com/article/us-cocoa-ivorycoast-idUSKCN1BO23M.

[CR2] Aboa, A. 2019. Ivory Coast Acts to Avert Defaults on Cocoa Export Contracts This Season. https://www.reuters.com/article/cocoa-ivorycoast-estimate-idUSL5N22C3N8.

[CR3] Aboa, A. 2021. Top Cocoa Producers Threaten to Name and Shame Brands Over Premiums. https://www.reuters.com/article/ivorycoast-ghana-cocoa-idUSL5N2O3518.

[CR4] Aboa, A., and M. Angel, M. 2019. Chocolate Makers Hobble Ivory Coast, Ghana Cocoa Premium with Discounts. https://www.reuters.com/article/us-cocoa-premiums/chocolate-makers-hobble-ivory-coast-ghana-cocoa-premium-with-discounts-idUSKBN1YL1W7.

[CR5] Akiyama, T., J. Baffes, D. Larson, and P., Varangis. 2001, eds. Commodity Market Reforms: Lessons of Two Decades, World Bank Regional and Sectoral Studies.

[CR6] Alence R (2001). Colonial Government, Social Conflict and State Involvement in Africa’s Open Economies: The Origins of The Ghana Cocoa Marketing Board, 1939–46. The Journal of African History.

[CR7] Amanor KS (2012). Global Resource Grabs, Agribusiness Concentration and the Smallholder: Two West African Case Studies. The Journal of Peasant Studies.

[CR8] Araujo Bonjean, C., and J. Brun. 2016. Concentration and Price Transmission in the Cocoa-Chocolate Chain. In: Squicciarini, Mara P./Swinnen, Johan (Eds.): The Economics of Chocolate. Oxford.

[CR9] Aulerich NM, Irwin SH, Garcia P (2013). Returns to Individual Traders in Agricultural Futures Markets: Skill or Luck?. Applied Economics.

[CR10] Baffes J, Etienne XL (2016). Analysing Food Price Trends in the Context of Engel’s Law and the Prebisch-Singer Hypothesis. Oxford Economic Papers.

[CR11] Bargawi HK, Newman SA (2017). From Futures Markets to the Farm Gate: A Study of Price Formation along Tanzania’s Coffee Commodity Chain. Economic Geography.

[CR12] Bauer PT (1954). Origins of the Statutory Export Monopolies of British West Africa. Business History Review.

[CR13] Beckert J (2011). Where Do Prices Come from? Sociological Approaches to Price Formation. Socio-Economic Review.

[CR14] Benjamin D, Deaton A (1993). Household Welfare and the Pricing of Cocoa and Coffee in Côte d’lvoire: Lessons from the Living Standards Surveys. The World Bank Economic Review.

[CR15] Berg, A. 2011. The Rise of Commodity Speculation: From Villainous to Venerable. FAO Commodity Market Review.

[CR16] Burch D, Lawrence G (2009). Towards a Third Food Regime: Behind the Transformation. Agriculture and Human Values.

[CR17] C̦alıșkan, K. 2010. Market Threads: How Cotton Farmers and Traders Create a Global Community. Princeton, NJ.

[CR18] Capelle-Blancard G, Coulibaly D (2011). Index Trading and Agricultural Commodity Prices: A Panel Granger Causality Analysis. International Economics.

[CR19] CCC. 2021. LE CONSEIL DU CAFE-CACAO FIXE LE PRIX BORD CHAMP DU CACAO À 825 FCFA /KG. http://www.conseilcafecacao.ci/index.php.

[CR20] CFTC. 2021. Commitment of Traders – Historically Compressed. https://www.cftc.gov/MarketReports/CommitmentsofTraders/HistoricalCompressed/index.htm.

[CR21] Clapp J (2014). Financialization, Distance and Global Food Politics. The Journal of Peasant Studies.

[CR22] Clarence-Smith WG (2000). Cocoa and Chocolate, 1765–1914.

[CR23] Close FA (1959). High Finance in Cocoa. Financial Analysts Journal.

[CR24] Dand R (2011). The International Cocoa Trade.

[CR25] COCOBOD. 2017. Response to the Minority Press Statement on Cocoa.

[CR26] COCOBOD. 2019. Côte D’Ivoire – Ghana Cooperation in Cocoa. https://cocobod.gh/news_details/id/204/PRESS%20RELEASE%20-%20CÔTE%20D’IVOIRE%20-%20GHANA%20COOPERATION%20IN%20COCOA.

[CR27] Ederer S, Heumesser C, Staritz C (2016). Financialization and Commodity Prices – An Empirical Analysis for Coffee, Cotton, Wheat and Oil. International Review of Applied Economics.

[CR28] Engel J, Avgouleas Emilios, Donald David C (2019). The Politics of Commodity Derivatives Reform in the EU and the USA. The Political Economy of Financial Regulation.

[CR29] Erten B, Ocampo JA (2013). Super Cycles of Commodity Prices Since the Mid-nineteenth Century. World Development.

[CR30] Eurostat. 2021. International Trade in Goods Database. https://ec.europa.eu/eurostat/web/international-trade-in-goods/data/database.

[CR31] FAO and BASIC. 2020. Comparative study on the distribution of value in European chocolate chains. Research Report - Advance Copy. Rome; Paris: Food; Agriculture Organization of the United Nations (FAO); Bureau d’Analyse Societale pour une Information Citoyenne (BASIC). https://lebasic.com/wp-content/uploads/2020/07/BASIC-DEVCO-FAO_Cocoa-Value-Chain-Research-report_Advance-Copy_June-2020.pdf.

[CR32] FCC (2019). Implementation of Living Income Differential by Côte d’Ivoire and Ghana.

[CR33] Fold N (2002). Lead Firms and Competition in ‘Bi-polar’ Commodity Chains: Grinders and Branders in the Global Cocoa-chocolate Industry. Journal of Agrarian Change.

[CR34] Fold, N., and J. Neilson. 2016. Sustaining Supplies in Smallholder Dominated Value Chains: Corporate Governance of the Global Cocoa Sector. The Economics of Chocolate, 195–20212.

[CR35] Fountain, A., and F. Hütz-Adams. 2018. Cocoa Barometer 2018. https://www.voicenetwork.eu/wp-content/uploads/2019/07/2018-Cocoa-Barometer.pdf. Accessed 14 Nov 2019.

[CR36] Gereffi G, Humphrey J, Kaplinsky R, Sturgeon TJ (2001). Introduction: Globalisation, Value Chains and Development. IDS Bulletin.

[CR37] Gibbon P (2014). Trading Houses During and Since the Great Commodity Boom: Financialization, Productivization or…?. DIIS Working Paper.

[CR38] Gibbon P (2001). Upgrading Primary Production: A Global Commodity Chain Approach. World Development.

[CR39] Gibbon P, Ponte S (2005). Trading Down. Africa, Value Chains, and the Global Economy.

[CR40] Gilbert, C.L. 1996. Informed Futures Market Speculation: Coffee and Cocoa, 1993–1995. SSRN 8455.

[CR41] Gilbert CL (2009). Cocoa Market Liberalization in Retrospect. Review of Business and Economic Literature.

[CR42] Guilleminot B, Ohana J, Ohana S (2014). The Interaction of Speculators and Index Investors in Agricultural Derivatives Markets. Agricultural Economics.

[CR43] Gombeaud J, Moutout C, Smith S (1990). La Guerre du Cacao. Histoire Secrète d’un Embargo.

[CR44] Grumiller J (2018). Upgrading Potentials and Challenges in Commodity-Based Value Chains: The Ivorian and Ghanaian Cocoa Processing Sectors. Journal Für Entwicklungspolitik.

[CR45] Haase M, Zimmermann YS, Zimmermann H (2016). The Impact of Speculation on Commodity Futures Markets–A Review of the Findings of 100 Empirical Studies. Journal of Commodity Markets.

[CR46] Hallam, D. 2017. Revisiting Prebisch-Singer: What Long-Term Trends in Commodity Prices Tell Us About the Future of CDDCs. Background Paper to the UNCTAD-FAO Commodities and Development Report.

[CR47] Hecht RM (1983). The Ivory Coast Economic 'Miracle': What Benefits for Peasant Farmers?. The Journal of Modern African Studies.

[CR48] Hieronymus TA (1977). Maipulation in Commodity Futures Trading: Toward a Definition. Hofstra l. Rev..

[CR49] Hütz-Adams, F., C. Huber, I. Knoke, P. Morazán, and M. Mürlebach. 2016. Strengthening the Competitiveness of Cocoa Production and Improving the Income of Cocoa Producers in West and Central Africa. SÜDWIND e.V., Bonn.

[CR50] ICCO. 2020. Quarterly Bulletin of Cocoa Statistics, Volume XLVI No. 3, Cocoa Year 2019/20.

[CR51] ICCO. 2021. ICCO Monthly Cocoa Market Report, October 2021. https://www.icco.org/app/statistics/#tab-id-1

[CR52] ICE. 2021. ICE Report Center https://www.theice.com/marketdata/reports/122

[CR53] IMF. 2019. Ghana. IMF Country Report Nr. 19/97. Washington DC.

[CR54] Isham J, Woolcock M, Pritchett L, Busby G (2005). The Varieties of the Resource Experience: How Natural-Resource Export Structures Affect the Political Economy of Economic Growth. World Bank Economic Review.

[CR55] ITC (2001). Cocoa: A Guide to Trade Practices.

[CR56] Kofi TA (1976). MNC Control of Distributive Channels: A Study of Cocoa Marketing. Stanford Journal of International Studies.

[CR57] Kolavalli, S., and M. Vigneri. 2017. The Cocoa Coast: The Board-Managed Cocoa Sector in Ghana. Washington DC.

[CR58] KPMG. 2018. Audit du systéme de commercialisation du cacao (Rapport pour Ministére de l’Agriculture et du Développement Rural).

[CR59] Labys WC, Thomas HC (1975). Speculation, Hedging and Commodity Price Behaviour: An International Comparison. Applied Economics.

[CR60] Levai LD, Meriki HD, Adiobo A, Awa-Mengi S, Akoachere JTK, Titanji VPK (2015). Postharvest Practices and Farmers’ Perception of Cocoa Bean Quality in Cameroon. Agriculture & Food Security.

[CR61] Losch B (2002). Global Restructuring and Liberalization: Côte d’Ivoire and the End of the International Cocoa Market?. Journal of Agrarian Change.

[CR62] Mallaby S (2010). More Money than God.

[CR63] McFarlne, S., and N. Hunt. 2015. Olam Completes Acquisition of ADM’s Cocoa Business. https://www.reuters.com/article/us-cocoa-olam-intl-archer-daniels-idUSKCN0SA24Y20151016.

[CR64] Murphy, S., D. Burch, and J. Clapp. 2012. Cereal Secrets: The world’s largest grain traders and global agriculture. Oxfam Research Report 80.

[CR65] Newman SA (2009). Financialization and Changes in the Social Relations Along Commodity Chains: The Case of Coffee. Review of Radical Political Economics.

[CR66] Nissanke M (2019). Exploring Macroeconomic Frameworks Conducive to Structural Transformation of sub-Saharan Economies. Structural Change and Economic Dynamics.

[CR67] Nkouedjo LL, Mathe S, Fon DE, Geitzenauer M, Manga AA (2020). Cocoa Marketing Chain in Developing Countries: How Do Formal-Informal Linkages Ensure Its Sustainability in Cameroon?. Geoforum.

[CR68] Ocampo JA (2017). Commodity-Led Development in Latin America. International Development Policy.

[CR69] ONCC. 2022. Office National du Cacao et du Café, Cameroon. Cocoa Statistics. https://oncc.cm/cocoa-statistics.

[CR70] Oomes, N., B. Tieben, A. Laven, T. Ammerlaan, R. Appelman, C. Biesenbeek, and E. Buunk. 2016. Market Concentration and Price Formation in the Global Cocoa Value Chain, SEO Amsterdam Economics.

[CR71] Poelmans, E., and J. Swinnen. 2016. A Brief Economic History of Chocolate. In Squicciarini, M.P., and J. Swinnen (Eds.) The Economics of Chocolate, 195–20212.

[CR72] Prebisch, R. 1950. The Economic Development of Latin America and Its Principal Problems. UN ECLA, Economic Bulletin for Latin America 7, United Nations, New York.

[CR73] Purcell TF (2018). ‘Hot Chocolate’: Financialized Global Value Chains and Cocoa Production in Ecuador. The Journal of Peasant Studies.

[CR74] Quarmine W, Haagsma R, Huis A, Sakyi-Dawson O, Obeng-Ofori D, Asante FA (2014). Did the Price-Related Reforms in Ghana’s Cocoa Sector Favour Farmers?. International Journal of Agricultural Sustainability.

[CR75] Reuters. 2021. Ivory Coast lowers 2020/21 Mid-crop Cocoa Farmgate Price by 9%. https://www.reuters.com/article/cocoa-ivorycoast-idUSL8N2LT1OE.

[CR76] Ross C (2014). The Plantation Paradigm: Colonial Agronomy, African Farmers, and the Global Cocoa Boom, 1870s–1940s. Journal of Global History.

[CR77] Salerno T (2016). Cargill’s Corporate Growth in Times of Crises: How Agro-commodity Traders Are Increasing Profits in the Midst of Volatility. Agriculture and Human Values.

[CR78] Sanders DR, Irwin SH (2011). The Impact of Index Funds in Commodity Futures Markets: A Systems Approach. The Journal of Alternative Investments.

[CR79] Sanders DR, Irwin SH (2017). Bubbles, Froth and Facts: Another Look at the Masters Hypothesis in Commodity Futures Markets. Journal of Agricultural Economics.

[CR80] Singer HW (1950). The Distribution of Gains Between Investing and Borrowing Countries. American Economic Review.

[CR81] Staritz C, Newman S, Tröster B, Plank L (2018). Financialization and Global Commodity Chains: Distributional Implications for Cotton in Sub-Saharan Africa. Development and Change.

[CR82] Talbot JM (2002). Tropical Commodity Chains, Forward Integration Strategies and International Inequality: Coffee, Cocoa and Tea. Review of International Political Economy.

[CR83] Trafigura. 2019. Commodities Demystified – A Guide to Trading and the Global Supply Chain. Report for Trafigura, Second Edition.

[CR84] UN Comtrade. 2021 UN Comtrade Database. https://comtrade.un.org/. Accessed 23 Nov 2021.

[CR85] UNCTAD/FAO. 2017. Commodities and Development Report 2017: Commodity Markets, Economic Growth and Development. United Nations Conference on Trade and Development, Geneva.

[CR86] Van Huellen, S. 2015. Excess Volatility or Volatile Fundamentals? The Impact of Financial Speculation on Commodity Markets and Implications for Cocao Farmers in Ghana. SOAS, University of London.

[CR87] Van Huellen S, Abubakar FM (2021). Potential for Upgrading in Financialised Agri-food Chains: The Case of Ghanaian Cocoa. The European Journal of Development Research.

[CR88] Varangis, P., and G. Schreiber. 2001. Cocoa Market Reforms in West Africa. In: Akiyama, Takamasa/Baffes, John/Larson, Donald/Varangis, Panos (Eds.) Commodity Market Reforms: Lessons of Two Decades, World Bank Regional and Sectoral Studies.

[CR89] Whitfield L (2018). Economies After Colonialism: Ghana and the Struggle for Power.

[CR90] Wimmer T, Geyer-Klingeberg J, Hütter M, Schmid F, Rathgeber A (2021). The Impact of Speculation on Commodity Prices: A Meta-Granger Analysis. Journal of Commodity Markets.

[CR91] World Bank. 2017. Ghana: Agriculture Sector Policy Note – Transforming Agriculture for Economic Growth, Job Creation and Food Security (Nr. AFR01). Agriculture Global Practice. https://openknowledge.worldbank.org/handle/10986/28394

