# Supplementary material for: Price-Setting Power in Global Value Chains: The Cases of Price Stabilisation in the Cocoa Sectors in Côte d’Ivoire and Ghana
Source: Eur J Dev Res. 2022 Jun 23:1–29. Online ahead of print. doi: 10.1057/s41287-022-00543-z (PMC9219397; doi:10.1057/s41287-022-00543-z)
Supplement: Supplementary file 1 — Supplementary file1 (DOCX 26 KB) [file 41287_2022_543_MOESM1_ESM.docx]

**Appendix I: Background information on interview partners**

| **Type of interview partners** | **Number of interviews** | **Location of interviews*** | **Time of interviews** |
| --- | --- | --- | --- |
| Grinder-traders and traders | 14, including global grinder-traders and locally-owned firms in producer countries | CH, UK (online), DE (online), GHA, CIV | Feb 2018 - Sep 2019 |
| Chocolate manufacturers | 5, including global chocolate manufacturers and manufacturers in producer countries | CH (online), GHA, CIV | Oct 2017 – Sep 2018 |
| Financial investors | 6, including investment banks and brokers | UK, CIV | Oct 2017 - Apr 2018 |
| International cocoa sector associations | 2 | UK, CIV | Jan 2019 - Apr 2018 |
| Sector experts | 8, including research institutes, donor organisations and NGOs | CH, NL (online), DE (online), GHA, CIV | Jan 2017 - Feb 2022 |
| Sector parastatals | 3 | GHA, CIV | Oct 2017 - Sep 2019 |
| Sector associations | 2, including exporter associations | CIV | Oct 2017 - Nov 2017 |
| Producer organisations | 7, including cocoa farmer associations and cooperatives | GHA, CIV | Jan 2017 - Sep 2019 |

* If not indicated as online, interviews were conducted in person.

**Appendix II: Thematic blocks of interviews**

| **Main topics** | **Subtopics** |
| --- | --- |
| Prices and price-setting | - Price levels and volatility - Intra- and inter-seasonal price volatility - Price-setting practices, strategies and institutions - Different actors’ price-setting power - Distribution of price risks - Price risk management - Dynamic of and activities on derivatives markets - Financialisation of derivatives markets |
| Cocoa GVC | - Actors and business strategies - Inter-firm relations and governance - Role of grinder-traders - Interrelation of physical and financial activities and strategies - Role of key producer countries - Income of cocoa producers |
| Regulation | - Cocoa sector regulation - Internal marketing systems - External marketing systems - Price stabilisation mechanisms - Resilience and challenges of price stabilisation - Living Income Differential - Recent reforms and challenges |
| Cocoa processing and chocolate manufacturing | - Global market developments - Producer country market developments - Competition between actors - Dynamics and challenges of producer country cocoa processing - Industrial policies targeting cocoa processing - Pricing of semi-processed cocoa products |

**Appendix III: List of Abbreviations**

AFFC Association Française du Commerce des Cacao

ANADER Agence Nationale d'Appui au Développement Rural

CAISTAB Caisse de stabilisation

CAL Cocoa Association of London

CCC Conseil du Café-Cacao

CFA West African CFA Franc

CIF Cost, Insurance, Freight

CIV Côte d’Ivoire

CMC Cocoa Marketing Company

CMMA Cocoa Merchants’ Association of America

COCOBOD Ghana Cocoa Board

COPAL Alliance of Cocoa Producing Countries

CTHs Commodity Trading Houses

EUR Euro

FCC Federation of Cocoa Commerce

FOB Free on Board

GBP British Pound Sterling

GHA Ghana

GHC Ghanaian Cedi

GVC Global Value Chain

ICAs International Commodity Agreements

ICCA International Cocoa Agreement

ICCO International Cocoa Organization

ICE Intercontinental Exchange

IMF International Monetary Fund

LBC Licensed Buying Company

LID Living Income Differential

PBC Produce Buying Company

PMPU Producer/Merchant/Processor User

PPRC Producer Price Review Committee

PTBF Price-to-be-fixed

QCC Quality Control Company Limited

SAP Structural Adjustment Programme

SF Stabilisation Fund

SSA Sub-Saharan Africa

USD United States Dollar
